# High nitrogen concentration alter microbial community in *Allium fistulosum* rhizosphere

**DOI:** 10.1371/journal.pone.0241371

**Published:** 2020-11-20

**Authors:** Chen Zhao, Haifeng Ni, Lin Zhao, Lin Zhou, Orlando Borrás-Hidalgo, Rongzong Cui

**Affiliations:** 1 Shandong Provincial Key Laboratory of Food and Fermentation Engineering, Shandong Food Ferment Industry Research & Design Institute, Qilu University of Technology (Shandong Academy of Sciences), Jinan, China; 2 State Key Laboratory of Biobased Material and Green Papermaking, Shandong Provincial Key Lab. of Microbial Engineering, Qilu University of Technology (Shandong Academy of Sciences), Jinan, China; 3 Institute of Agricultural Resources and Environment, Shandong Academy of Agricultural Sciences, Jinan, China; Agroecological Institute, CHINA

## Abstract

Welsh onion (*Allium fistulosum* L.) constitutes an important plant species cultivated in China due the benefits and applications in different areas. Moreover, nitrogen is an essential nutrient during the growth and development of plant. Here, we present the effects of nitrogen on soil microbiome in welsh onion plants. We used High-throughput sequencing analysis to determine the diversity and abundances of microbes associated to soil rhizosphere in welsh onion under the influence of nitrogen application. Nitrogen application significantly influenced in the diversity of fungal community. The relative abundance of Orbiliomycetes increased with the nitrogen concentration. Nitrogen application did not affect the diversity of bacterial community, whereas the relative abundance of Acidobacteria_Gp2, Verrucomicrobiae and Sphingobacteriia decreased with the nitrogen condition. In this work, we introduced evidences of the effect of nitrogen fertilization on microbial community in welsh onion rhizosphere, and the change of microbial community may interfere the growth and development of welsh onion.

## Introduction

Welsh onion (*Allium fistulosum* L.) has a great importance in China and abroad. This plant is rich in carbohydrates, proteins, minerals and vitamins. Additionally, this contains propylene sulfide with antibacterial and anti-inflammatory effects [1–3]. Microbial community structure has unique responses to various biotic and abiotic conditions [1–4]. Such as Flavobacterium species recovered from the rhizosphere soils of Allium plants play roles in inhabiting in *Fusarium* wilt suppression [5]. Previous studies have showed as some environment variables of soil including pH, dissolved organic carbon, nitrogen and vegetation were major determinants of microbial composition, diversity, and richness in ecosystems [6–8].

Nitrogen is one of the most important mineral elements in plants. The available nitrogen in the soil was the major contributor to plant growth and productivity [9]. Nitrate and ammonium are the universal forms acquired for plants in the soil. Large amounts of nitrogen fertilizers were applied over the past few decades in China [10]. However, excessive application of nitrogen fertilizer does not always lead to a continuous increase in crop yields [11]. In natural soil, availability of nitrogen was generally diverse, depending on various factors including soil physical properties and microbial communities [12,13]. On the other hand, nitrogen is one of the most important nutrients for the growth and development of welsh onion [14]. Microbial communities played significant roles in promoting nitrogen uptake in plants. Application of nitrogen resulted in changes of bacterial community composition and richness in surface soil [15]. In addition, bacterial community composition was also significantly affected by plant composition [15]. Many studies on soil microbial communities in different ecosystems were reported [16–18]. However, studies on microbial communities in welsh onion rhizosphere during the application of different nitrogen fertilizer were not developed, previously. Recently, knowledge of composition and functions of microbial communities has increased significantly with the development of DNA sequencing and metagenomics [19,20]. For example, endophytic bacterial diversity of four Allium species was analyzed by Illumina MiSeq sequencing [21]. Flavobacterium species from rhizosphere soils of Allium plants suppressed *Fusarium* wilt were determined by Illumina MiSeq sequencing [5]. In this study, we used the High-throughput sequencing to screening the microbial abundance and community changes in response to different nitrogen fertilization in the rhizosphere soil of welsh onion. The main objective was the evaluation of the correlation between the welsh onion growth with the microbial community by analyzing the effect of nitrogen, and further enrich the mechanism of nitrogen promoting the growth in welsh onion.

## Materials and methods

### Samples collection

Soil samples were collected from different nitrogen application experiments conducted in the experimental field belonging to Shandong Academy of Sciences at the Zhangqiu experimental field, Jinan City, Shandong Province, China (36˚72ˈ N, 117˚53ˈ E). The region has a temperate sub-humid continental monsoon climate, with a mean annual temperature of 12.6°C and average annual precipitation of 600.8 mm. The soil type is classified as cinnamon soil. The soil nitrogen concentration was at 80.67mg kg^−1^ prior to the experiment. The nitrogen addition experiment was established during June 2017 when welsh onion was planted. Three nitrogen concentration were evaluated, including a control without nitrogen application. Thus, the treatment was the following: control (0 kg ha^-1^), half N (130kg ha^-1^) and full N (260kg ha^-1^). Nitrogen fertilizer was applied at four time (June 25, August 14, August 27, September 9), respectively. The treatments (0 N, half N, full N) were named as: N0, N1, and N2.

A total of 15 micro-areas were set in the micro-zone test. Zero, half and full nitrogen fertilizer application were prepared from south to north in the micro-zone with five replications. Each micro-area was 80 × 100 cm. The micro-areas were separated by aisles files from 1 meter. On November 13, 2017, welsh onion was harvested. For each nitrogen treatment, 10 plants were randomly selected in the micro-areas. Plant shoot length, pseudostem length, pseudostem diameter, fresh weight of pseudostem, fresh weight of leaf, fresh weight of root, dry weight of leaf, dry weight of pseudostem and dry weight of root were evaluated, and the average value of 10 plants was used as the measured value for each treatment. After welsh onion being harvested, soil was collected from each treatment. The rhizosphere soil within 3 cm near the welsh onion root was collected. Soil samples were passed through a 2 mm sieve, fully homogenized. One portion of the homogenized soil will be used for soil DNA extraction. Each treatment chose three replications for further analysis.

### DNA extraction and High-throughput sequencing

Total microbial genomic DNA was extracted from 0.5 g above homogenized soil by using a fast E.Z.N.A^TM^ Mag-Bind Soil DNA Kit (OMEGA, USA). The quantity and quality of the extracted DNA samples were measured by Qubit® 3.0 Fluorometer (Invitrogen, USA). To determine bacterial and fungal community composition and diversity in the soil samples, Illumina MiSeq High-throughput sequencing was done. The V4-V5 hypervariable region of the bacterial 16S rRNA was amplified using primers 515F (5′-GTGCCAGCMGCCGCGG-3′) and 907R (5′CCGTCAATTCMTTTRAGTTT-3′) [10]. The fungal ITS region of fungal rRNA was amplified using primers ITS1F (5′-GTGCCAGCMGCCGCGG-3′) and 2043R (5′-GCTGCGTTCTTCATCGATGC-3′) [10]. Both primers were tagged with a unique barcode sequence (8 mer) to each sample. PCR of each sample was carried out in a 20 μL reaction system containing 10 ng template DNA, 4 μL 5 × FastPfu Buffer, 0.8 μL of 5 μM forward primer, 0.8 μL of 5 μM reverse primer, 2 μL of 2.5 mM dNTPs, and 0.4 μL TransStart FastPfu DNA Polymerase (Transgen, China). The PCR conditions for the bacterial 16S rRNA and fungal ITS rRNA were as follows [10]: denaturation at 95 °C for 3 min, 27 cycles (33 cycles for ITS) of 95 °C for 30 s, 55 °C for 30 s, 72 °C for 45 s, and final extension at 72 °C for 10 min. PCR products for each sample were purified by an MagicPure Size Selection DNA Beads (Transgen, Beijing, China). The purified PCR products from different samples were pooled using equimolar amounts to obtain a quantitative sample DNA library, and then used for sequencing using an Illumina MiSeq platform at the Sangon Biotech (Shanghai) Co., Ltd. Shanghai, China.

### Sequencing data analysis

Raw sequences were analyzed by using the Quantitative Insights Into Microbial Ecology (QIIME) toolkit (version 1.8.0) [22]. All reads were trimmed, merged and assigned in QIIME. The primers and low-quality sequences with read shorter than 200bp and with an average quality score lower than 20 were removed. Operational taxonomic units (OTUs) were then clustered at the 97% sequence similarity level using the U search program (version 5.2.236). The most abundant sequence for each OTU was selected as the representative OUT for various OTU analyses. Each OTU’s representative sequence was aligned using the Python Nearest Alignment Space Termination (PyNAST, version 1.2.2) against the Sliver 16S rRNA database for bacteria and Unite ITS database for fungi [23]. To estimate bacterial and fungal alpha diversity, diversity indices, OTU richness and Chao richness estimators were calculated by using the QIIME pipeline. Reads annotation was applied to create functional profiles searching against the COG database [24,25].

### Statistical analysis

For alpha diversity analysis, ACE/Chao/Shannon/Simpson/Richness indices were calculated in QIIIME [22]. For cluster tree analysis, the vegan package of R was used to calculate beta diversity distance matrix (hierarchical clustering) according to the species abundance of each sample. The method used for calculating the distance among samples is Bray Curtis. Then, the unweighted pair group method with arithmetic mean was used to construct the tree. For species abundance analysis, fisher exact test was used to obtain the P value. The P value was subjected to Multiple Test Correction using false discovery rate (FDR) to obtain a Q value.

## Results

### Welsh onion yields post different nitrogen application

The nitrogen fertilizer addition experiment was established when welsh onion plants were planted. The yields were significantly correlated with soil nitrogen content. The application significantly increased the yields of welsh onion (Table 1). This had the highest yields in the full N fertilizer treatment (N2) followed by half nitrogen fertilizer (N1). The welsh onion planted in soil without excess nitrogen fertilizer application (N0) had the lowest yields. The shoot length, fresh weight of pseudostem, fresh weight of leaf, fresh weight of root, and dry weight of leaf of welsh onion planted in soil with full nitrogen fertilizer were significantly higher than both of those with half nitrogen fertilizer and zero nitrogen fertilizer (Table 1). For N1 treatment, fresh weight of pseudostem, fresh weight of leaf, dry weight of leaf, and the shoot length of welsh onion were also significantly higher than those in N0 treatment (Table 1). However, there were not significant differences on pseudostem length, pseudostem diameter, dry weight of pseudostem and dry weight of root between full and half nitrogen fertilizer application (Table 1).

**Table 1:** Effects of different nitrogen levels on the physiological indexes of *Allium fistulosum*.

N treatment	Shoot length (cm)	Stalk length (cm)	Stalk diameter (mm)	Fresh weight of stalk (g)	Fresh weight of leaf (g)	Fresh weight of root (g)	Dry weight of stalk (g)	Dry weight of leaf (g)	Dry weight of root (g)
N0	115.7±7.37^a^	43.53±1.58^a^	18.6±1.02^c^	99.97±3.00^b^	86.5±10.11^b^	3.84±0.75^b^	7.99±0.54^a^	7.49±0.79^b^	0.898±0.20^b^
N1	115.9±1.96^a^	43.67±0.24^a^	23.21±0.36^b^	139.1±11.25^ab^	119.58±7.57^ab^	3.75±0.81^b^	10.68±0.48^a^	11.74±1.60^ab^	1.024±0.29^ab^
N2	124.43±5.15^a^	43.53±1.60^a^	24.91±0.62^a^	156.95±19.50^a^	167.31±23.95^a^	6.15±0.66^a^	11.87±1.64^a^	15.83±2.31^a^	1.3±0.25^a^

Data were performed in triplicates and represented as mean ± SD.

### Alpha diversity of soil microbial community

**Fig 1:**
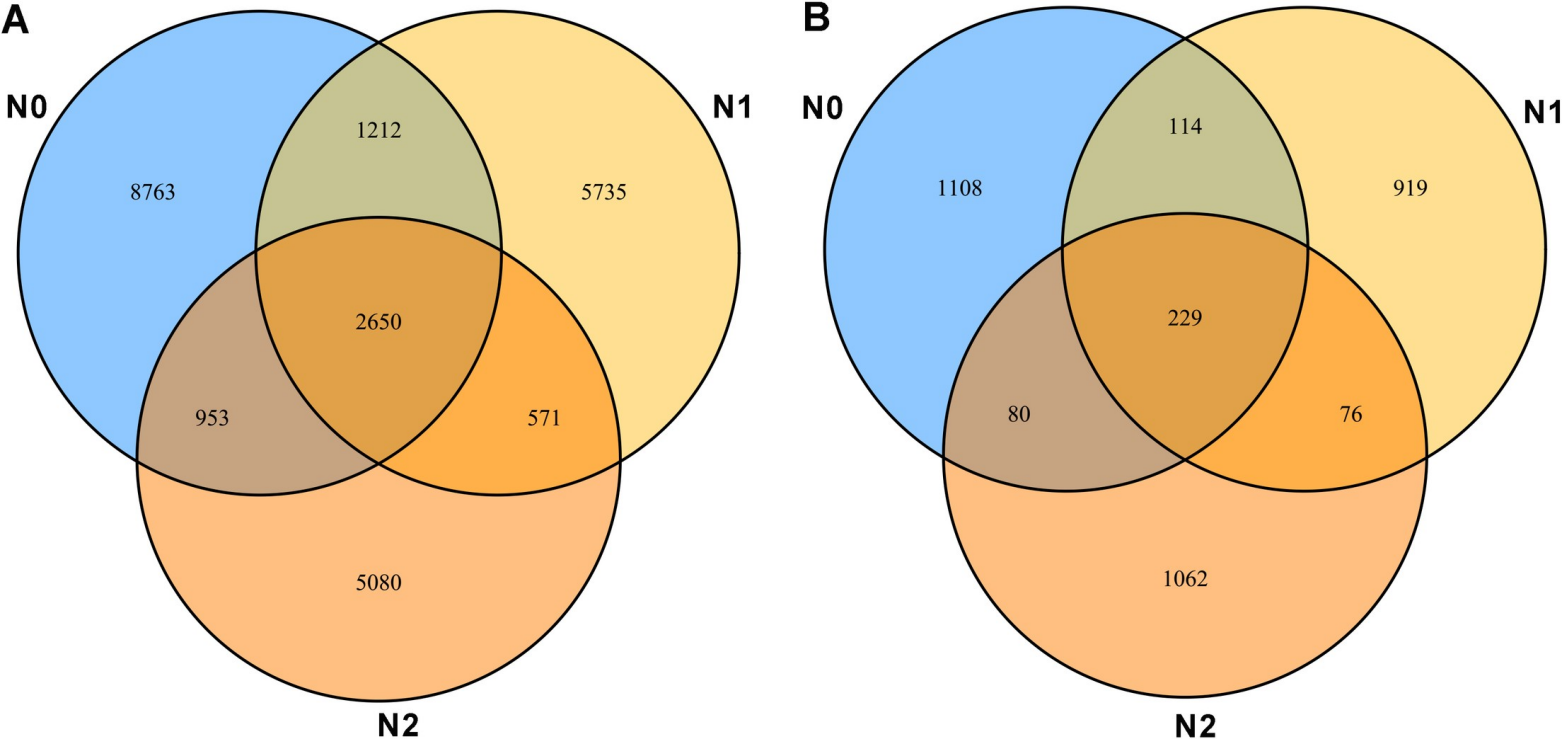
Venn map showing the number of detected bacterial and fungal OUTs found in soil samples with different nitrogen contents. (A) Venn map of the number of bacterial OUTs. (B) Venn map of the number of fungal OUTs. Blue, light yellow and dark yellow circles represent soil samples containing zero nitrogen (N0), half nitrogen (N1) and full nitrogen (N2), respectively. Overlapping parts of the circles represent the number of shared OUTs and non-overlapping parts represent the number of unique OUTs for each sample.

After quality filtering of raw data, totals of 168,758 (41,770–77,219 sequences per sample) and 159,110 (39,175–64,381 sequences per sample) high quality 16S rRNA and ITS sequences were obtained, respectively. A total of 28,552 operational taxonomic units (OTUs), including 24,964 and 3,588 OTUs of bacterial and fungal community in soil samples, were defined at 97% similarity among these sequences and were used for the subsequent analysis. The Venn diagram was used to count the number of common and unique OTUs in all samples, which shows the similarity and overlap of OTUs in the different soil samples. A Venn diagram based on OTUs comparing the three nitrogen addition treatments revealed that 10.62% and 6.38% OTUs of bacteria and fungi were shared by the three levels of nitrogen treatments (N0, N1, N2), respectively, whereas each nitrogen treated soil sample contained more special OTUs of bacteria and fungi (Fig 1). For bacterial community, the three nitrogen treatment shared 27 bacterial phyla, whereas, Woesearchaeota and Pacearchaeota were just exist in N2 treatment (Fig 1). For fungal community, there were four fungal phyla, including Ascomycota, Basidiomycota, Zygomycota, Chytridiomycota, shared by the three nitrogen treatments, whereas, Glomeromycota phyla was only found in N2 treatment (Fig 1). The Shannon index of bacterial community was similar in N0, N1 and N2 (7.66, 7.53 and 7.62) fertilizer application, and the Simpson indexes of bacterial community in the three nitrogen application were all zero. However, the Shannon index (3.67, 3.60, 4.44) of the fungal community was increased with the increasing of N fertilizer application (N0, N1, N2), and the Simpson index (0.11, 0.09, 0.03) was decreased with the increasing of N fertilizer application (N0, N1, N2).

### Bacterial and fungal composition at phyla and classes level

**Fig 2:**
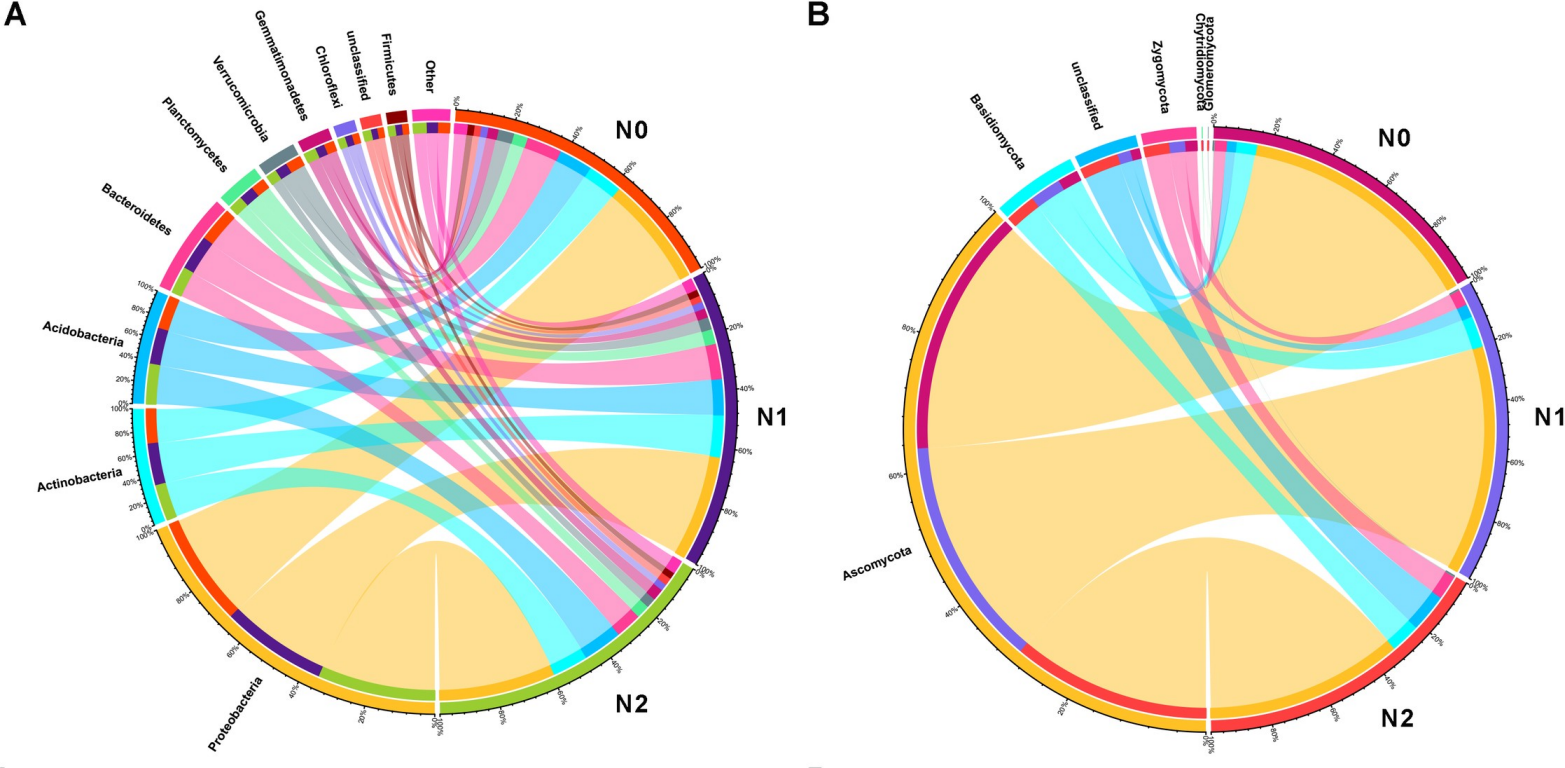
Relationship between different soil samples and microbial community at phylum level. (A) Collinearity diagram of bacterial communities with different soil samples. (B) Collinearity diagram of fungal communities with different soil samples. N0 represents zero nitrogen, N1 represents half nitrogen and N2 represents full nitrogen. Value on the outside circle indicates the abundance of the corresponding species.

The bacterial OTUs were assigned into 31 phyla (containing one unclassified group), 63 classes, 89 orders, 176 families and 559 genera. The fungal OTUs were assigned into 6 phyla (containing one unclassified group), 20 classes, 51 orders, 89 families and 157 genera. The overall composition of both bacterial and fungal phyla remained relatively stable in response to different nitrogen fertilization at the moment of harvest. The bacterial distribution of each phylum in different nitrogen treated soil samples was also similar (Fig 2). The dominant bacterial phyla were Proteobacteria, Actinobacteria, Acidobacteria, Bacteroidetes, Planctomycetes, Verrucomicrobia, Gemmatimonadetes, Chloroflexi, unclassified, Firmicutes and represented >95% of the total bacterial sequences. Proteobacteria was the most dominant, represented for 38.37%, 36.84% and 40.84% in N0, N1 and N2 soil samples, respectively (Fig 2). The distribution of each fungal phylum in soil samples with different nitrogen fertilizer application was different (Fig 2). Ascomycota and Basidiomycota were the two most dominant fungal phyla, with a 92%, 90% and 78% of the total fungal sequences in N0, N1 and N2 soil samples, respectively. With the increase of nitrogen addition, the proportion of Ascomycota was reduced, while Basidiomycota and Zygomycota were increased (Fig 2). Zygomycota was another dominant fungal phylum, which the ratio was increased with the increasing of nitrogen addition.

**Fig 3:**
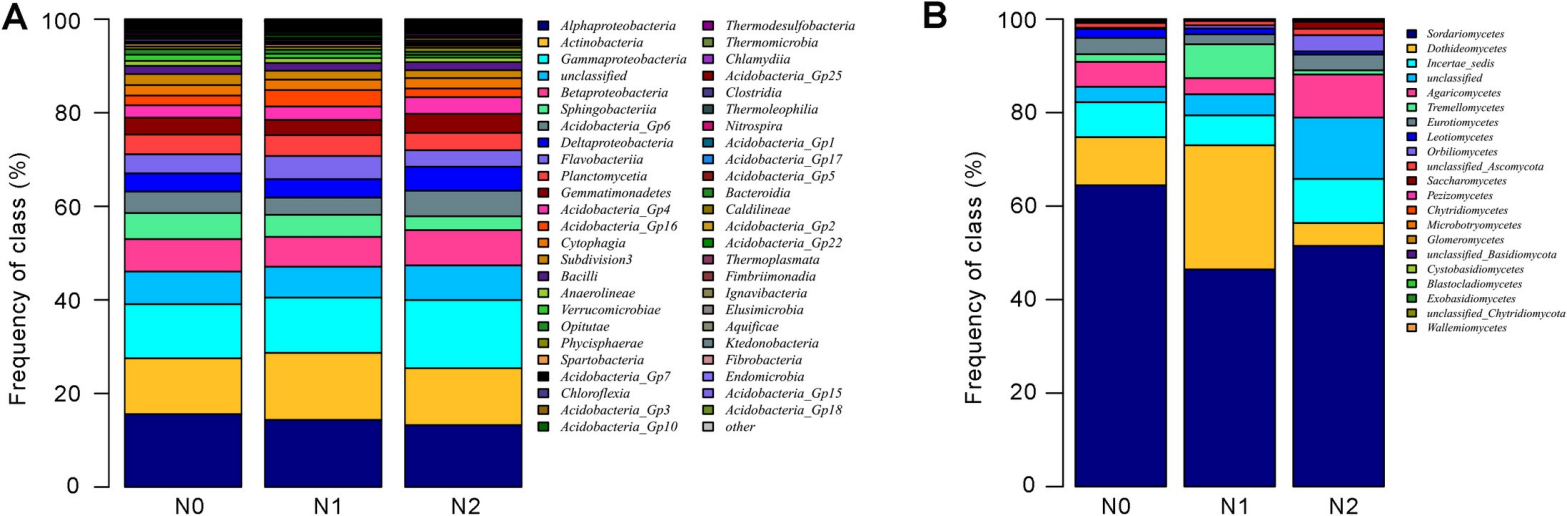
Relative abundance of dominant bacterial classes and fungal classes in soil samples with different nitrogen contents. (A) Relative abundance of dominant bacterial classes across different nitrogen fertilizer applications. (B) Relative abundance of dominant fungal classes across different nitrogen fertilizer applications. N0 represents zero nitrogen, N1 represents half nitrogen and N2 represents full nitrogen.

The Alphaproteobacteria (13.25%-15.59%), Actinobacteria (11.93%-14.32%), Gammaproteobacteria (11.58%-14.57%), were the three most abundant classes in all bacterial sequences (Fig 3). The relative abundance of Alphaproteobacteria and Sphingobacteriia were reduced in soil samples of N1 and N2 compared with the N0, whereas Gammaproteobacteria and Acidobacteria_Gp4 were increased (Fig 3). In addition, Acidobacteria_Gp2, Verrucomicrobiae and Sphingobacteriia were the three most declined classes in N1 and N2 compared with N0 (Fig 3). Several classed of fungi was affected by N-addition level (Fig 3). Sordariomycetes (51.50%-64.46%) was the most abundant class in all fungal sequences (Fig 3). The relative abundance of Sordariomycetes was lower in N1 (46.47%) and N2 (51.50%) than in N0 (64.46%). In contrast, the relative abundance of Orbiliomycetes belonging to saprophytic fungi was increased with the increase of nitrogen addition (Fig 3).

### Fungal composition at species level

**Fig 4:**
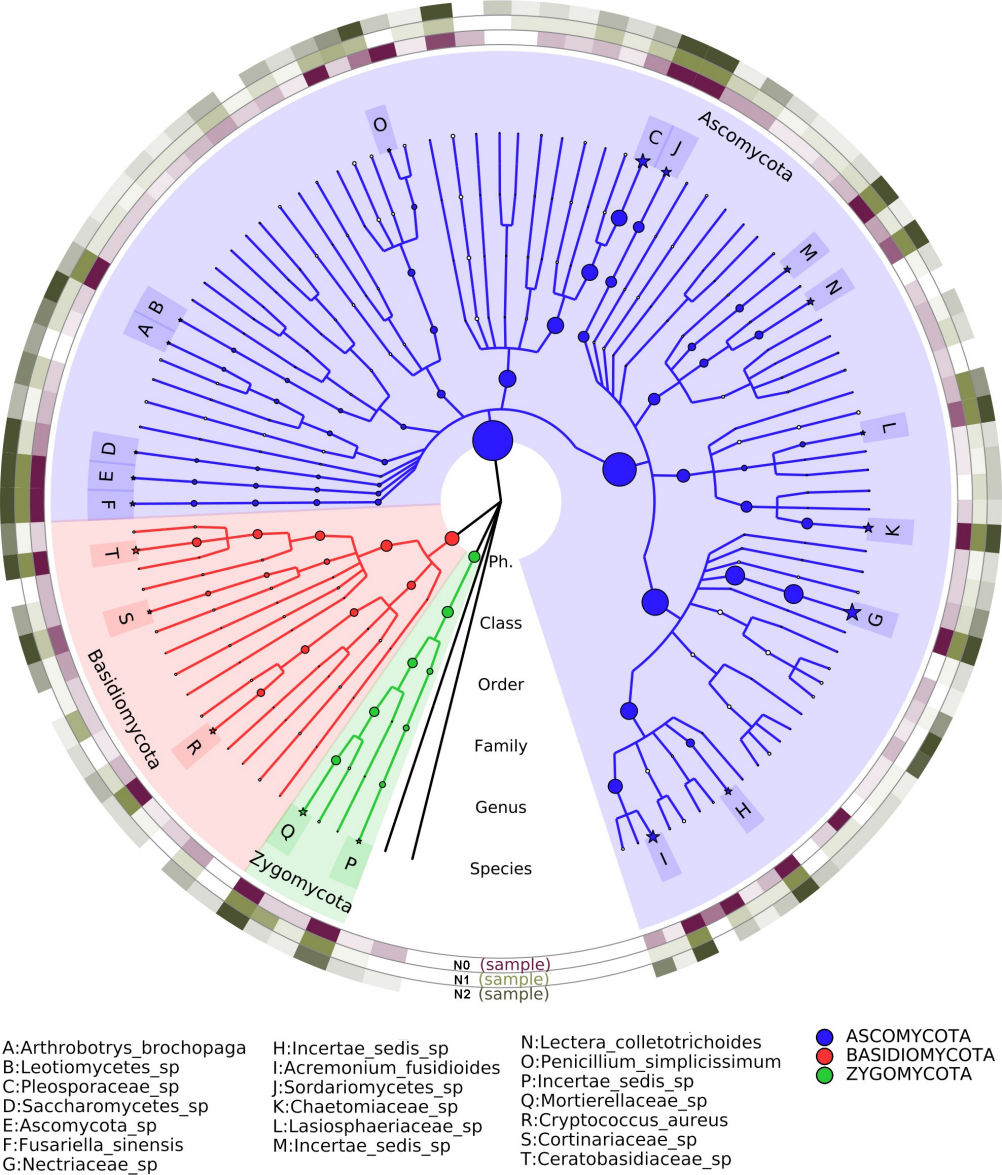
Different abundant taxa of fungi in soil samples with different N contents. From the center to the outside, they represent the kingdom, phylum, class, order, family, genus, and species. N0 represents zero nitrogen, N1 represents half nitrogen and N2 represents full nitrogen.

The nitrogen addition had more effect on fungal community, distribution of species belonging to phyla of Ascomycota, Basidiomycota and Zygomycota in different nitrogen treatments. Fifteen of the top 20 richest species belong to Ascomycota, two belong to Zygomycota, and three belong to Basidiomycota (Fig 4). The percentages of *Mortierellaceae*_sp and *Ceratobasidiaceae*_sp of Basidiomycota were higher in N2 (7.58% and 5.09%) treatment than in N0 (2.75% and 3.70%). The *Chaetomiaceae*_sp of Ascomycota was higher in N1 (5.49%) and N2 (7.72%) than in N0 (3.60%). In contrast, the percentage of seven species, including *Trichocomaceae*_sp of Zygomycota and six (*Leotiomycetes*_sp, *Nectriaceae*_sp, *Penicillium_rubidurum*, *Sarocladium_implicatum*, *Trichoderma_harzianum*, *Fusariella_sinensis*) of Ascomycota were lower both in N1 and N2 than in N0.

### Potential function analysis

**Fig 5:**
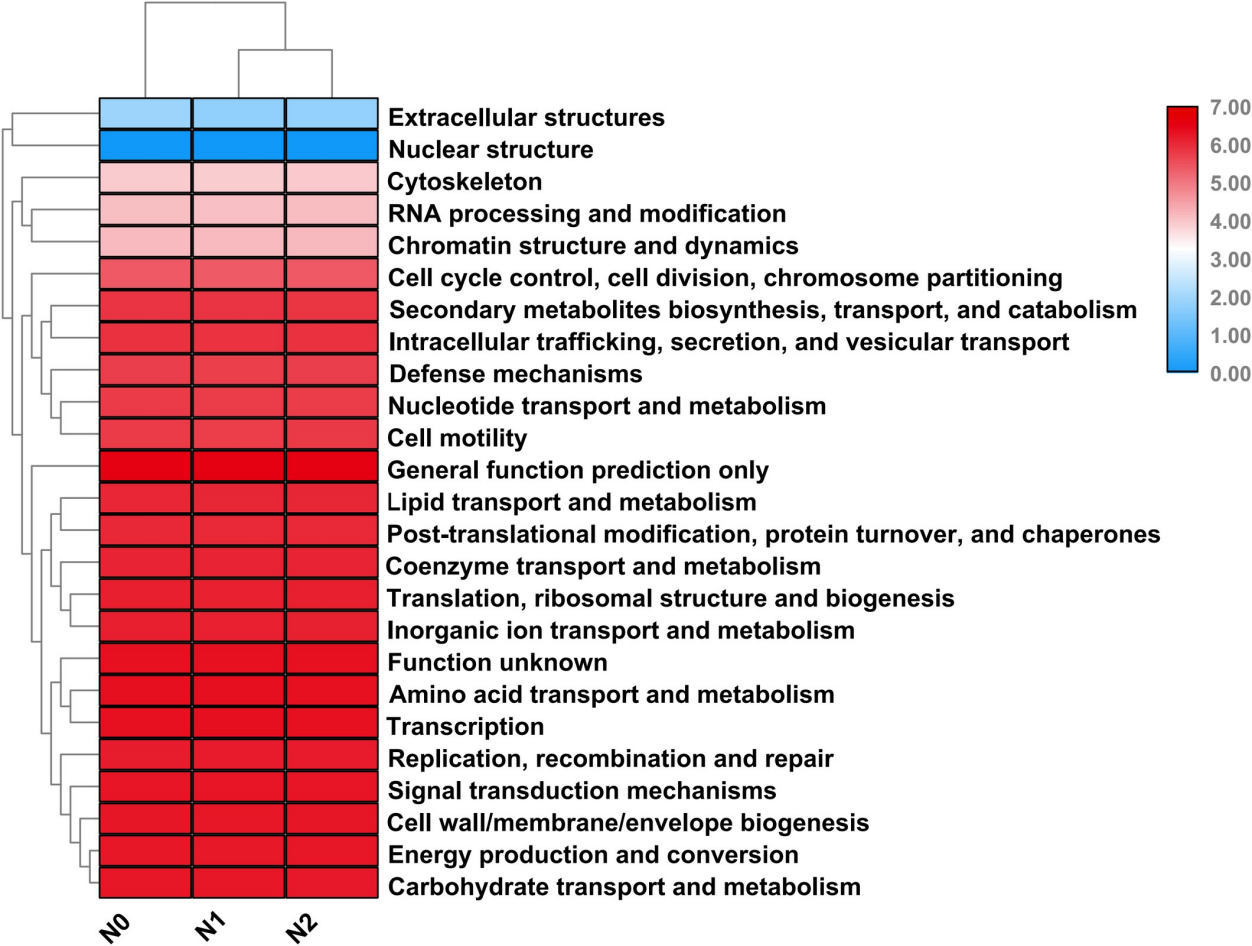
Relative abundance of COGs in metagenomic libraries of bacterial communities in soil samples with different nitrogen contents. The potential function determined by annotating metagenomic reads using COG database Level 2. Read counts of each functional group were normalized.

Functional gene composition in the sample was showed by comparing the functional analysis of High-throughput sequencing data and the corresponding 16S prediction function analysis. The potential functions of bacterial communities were determined by affiliating sequencing data into COG categories. Like bacterial taxonomic data, potential function data showed fewer differences among three nitrogen addition treatments when using COG categories at level 2. COG functions from 25 categories were identified (Fig 5), and all the categories had similar representation in different nitrogen addition soil samples.

## Discussion

The application of nitrogen fertilizer promoted the yields according previous results [26,27]. The total yield of onion (*Allium cepa* L.), a closed relative of the welsh onion, was increased significantly due to application of nitrogen fertilizer, while the yield was low without nitrogen application [28]. In the present study, nitrogen fertilizer also promoted welsh onion plants growth. Increasing of growth and yield of welsh onion might be due to an increasing of available nitrogen content in the soil post nitrogen fertilizer supply. In addition to soil nitrogen concentration can improve welsh onion plant growth, soil microorganisms may also play important roles. Thus, understanding changes to the composition of soil microorganisms in response to nitrogen fertilization is important for promoting welsh onion plant growth. In this study, we evaluated the changes of microbial community in the rhizosphere of welsh onion under different nitrogen application. This is the first time for these changes in microbial composition have been evaluated for welsh onion under different nitrogen conditions.

In the present study, the α-diversity analysis showed no effect of different nitrogen application on bacterial community composition, indicating the soil bacterial community composition were relatively stable under different nitrogen conditions. Off course, this phenomenon occurring may be due to the short treatment time, that the effects of nitrogen fertilizer on bacterial communities could not fully demonstrated. However, application of nitrogen fertilizer had some affection on fungal alpha-diversity. Application of full nitrogen fertilizer (N2) in soil showed higher fungal diversity (Shannon index 4.44 and Simpson index 0.03) than that of soil without excess nitrogen addition (N0, Shannon index 3.67 and Simpson index 0.11). While half nitrogen fertilizer application (N1) had not affection on fungal diversity when compared with N0. Unlike other reports, which Shannon diversity and richness were decreased with nitrogen fertilizer application in spruce forest [29], full nitrogen fertilizer application in welsh onion field increased fungal diversity. This difference between the current study and previous works might be resulted from the different species or basal nitrogen content in soil. Different plants had different nitrogen use rates and different dominant microbial community [30–33].

In our analysis, Proteobacteria, Actinobacteria, Acidobacteria, were the most dominant phyla in the rhizosphere of welsh onion. Interestingly, Proteobacteria is known as a specific symbionts of legume, and plays an important role in nitrogen fixation [34]. Some studies also reported that members of Actinobacteria demonstrate nitrogen-fixing function [35], and several species of Actinobacteria are involved in symbiotic nitrogen fixation [36]. Liu et al. (2017) showed that the Acidobacteria relative abundance was influenced by nitrogen dose and form in Chinese Fir Plantations [37], but it abundance was not effected by nitrogen concentration in this study. Above all, as the most abundance phyla, Proteobacteria, Actinobacteria and Acidobacteria may play the main roles in promoting nitrogen uptake for welsh onion plant that not depending on nitrogen concentration in soil.

Similar like fungal diversity in tea soils [38], Ascomycota, Basidiomycota and Zygomycota were the most dominant fungal phyla in different nitrogen addition soil samples, indicating the three phyla may play important roles in promoting nitrogen uptake that not depending on plant species. The abundance of Ascomycota and Basidiomycota were higher in N1 than N0 and N2, which was similar like Mueller *et al*. (2012) study, Ascomycota and Basidiomycota were increased at intermediate levels of nitrogen addition, but declined at higher levels [39], indicating the abundance of Ascomycota and Basidiomycota may be influenced by nitrogen concentration. In addition, Glomeromycota phyla was only found in N2 treatment of fungal community. Arbuscular mycorrhizal (AM) fungi is beloing to the Glomeromycota phyla, and it can form mutualistic associations with most plants in natural conditions [40]. It has been suggested that Glomeromycota are structured by soil factors [41,42]. The existence of Glomeromycota phyla in N2 suggested the nitrogen application of N2 is more suitable for Glomeromycota alive and development than N0 and N1. Additional, AM fungi play crucial roles in promoting nitrogen uptake for plant, the existence of Glomeromycota can promote nitrogen uptake for welsh onion. Although changes of microbial community in welsh onion rhizosphere in response to different nitrogen application have been revealed in this study, functions of those altered microbial species in welsh onion growth and development were not clear.

## Conclusions

Nitrogen application promoted growth and yield of welsh onion plants. The alpha-diversity of the fungal community was more sensitive to changes of application than bacterial community. In fungal community, the relative abundance of Orbiliomycetes belonging to saprophytic fungi was increased with the increasing of nitrogen application rates. In bacterial community, the relative abundance of Acidobacteria_Gp2, Verrucomicrobiae and Sphingobacteriia were reduced with the increasing of applications. The present study showed that higher nitrogen application had greater effect on microbial community in welsh onion rhizosphere.
