# Correction: High nitrogen concentration alter microbial community in *Allium fistulosum* rhizosphere

**DOI:** 10.1371/journal.pone.0246163

**Published:** 2021-01-22

**Authors:** Chen Zhao, Haifeng Ni, Lin Zhao, Lin Zhou, Orlando Borrás-Hidalgo, Rongzong Cui

An additional affiliation is missing for the first author. Chen Zhao is also affiliated with State Key Laboratory of Biobased Material and Green Papermaking, Shandong Provincial Key Lab. of Microbial Engineering, Qilu University of Technology (Shandong Academy of Sciences), Jinan, China.
